# An Immersive Investment Game to Study Human-Robot Trust

**DOI:** 10.3389/frobt.2021.644529

**Published:** 2021-06-04

**Authors:** Sebastian Zörner, Emy Arts, Brenda Vasiljevic, Ankit Srivastava, Florian Schmalzl, Glareh Mir, Kavish Bhatia, Erik Strahl, Annika Peters, Tayfun Alpay, Stefan Wermter

**Affiliations:** Knowledge Technology Group, Department of Informatics, Universität Hamburg, Hamburg, Germany

**Keywords:** human-robot interaction, investment game, non-verbal communication, science fiction, human-robot trust

## Abstract

As robots become more advanced and capable, developing trust is an important factor of human-robot interaction and cooperation. However, as multiple environmental and social factors can influence trust, it is important to develop more elaborate scenarios and methods to measure human-robot trust. A widely used measurement of trust in social science is the *investment game*. In this study, we propose a scaled-up, immersive, science fiction Human-Robot Interaction (HRI) scenario for intrinsic motivation on human-robot collaboration, built upon the investment game and aimed at adapting the investment game for human-robot trust. For this purpose, we utilize two Neuro-Inspired COmpanion (NICO) - robots and a projected scenery. We investigate the applicability of our space mission experiment design to measure trust and the impact of non-verbal communication. We observe a correlation of 0.43 (p=0.02) between self-assessed trust and trust measured from the game, and a positive impact of non-verbal communication on trust (p=0.0008) and robot perception for anthropomorphism (p=0.007) and animacy (p=0.00002). We conclude that our scenario is an appropriate method to measure trust in human-robot interaction and also to study how non-verbal communication influences a human’s trust in robots.

## 1 Introduction

As robot capabilities become more and more sophisticated, we not only want them to solve increasingly complex tasks independently but ultimately aid humans in their day-to-day life. Moreover, such social robots should act in a way that is reliable, transparent, and builds trust in their capabilities as well as their intentions (Felzmann et al., 2019). As soon as humans and robots autonomously work in a team on collaborative tasks, trust becomes essential for effective human-robot interaction (Casper and Murphy, 2003). This shows the need for a deeper understanding of what makes us willing to cooperate with robots and which factors enhance or destroy trust during interactions.

We approach this topic by adopting the investment game by Berg et al. (1995), a widely used experiment to measure trust in *human-human* collaboration. In the investment game, trust is measured as the amount of money a person is willing to give to an anonymous counterpart, in the prospect of a future profit. While others have used it in an HRI setting, some report limitations and differences when applying it to human-robot collaboration (which we elaborate on in Section 2). We, therefore, adapt the original investment game toward a persuasive HRI cooperative scenario by scaling up both the robotic agent as well as the environment. With scaling up we allude to the progression toward a human-like interaction: a realistic cooperative scenario as opposed to an abstract exchange of money. We do this by introducing a plausible currency for both humans as well as robotic agents, along with a weighted choice between two trustees, and removing the ability of the participant to make choices based on domain knowledge. The result is an HRI scenario, concealed as a futuristic, immersive spaceship adventure containing multiple rounds of the investment game for participants to develop intrinsic motivation to collaborate with robots.

**FIGURE 1:**
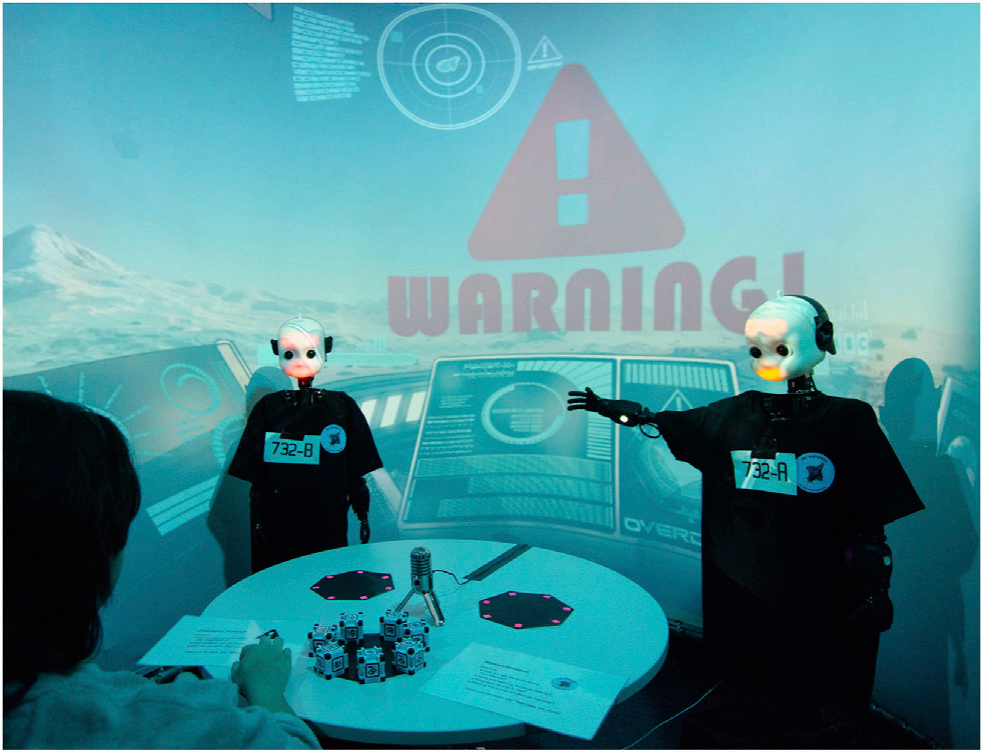
The experimental setup. On the table there are three compartments, with the one in the front (closest to the participant) containing the total amount of 7 energy cells to distribute.

In this scenario, we utilize two Neuro-Inspired COmpanion (NICO) humanoid robots by Kerzel et al. (2020) to advise the participant who acts as a spaceship commander. A voice-controlled artificial intelligence system which we refer to by “Wendigo” guides the participant through the experiment, where a large curved projector screen with an interactive video feed simulates the inside of the ship’s cockpit (see Figure 1). The setup is fully autonomous, with automatic speech recognition, visual detection as well as dialogue management implemented as ROS (Quigley et al., 2009) services, yet allows the experimenter to intervene on necessity. During the scenario, participants encounter four similar challenges (navigation malfunctioning, impending asteroids, engine failures and leaks in the cooling system): after the problem is announced by the ship AI, the robotic advisers propose two diverging solutions. Subsequently, the participants are asked to make a choice by distributing the ship’s energy resources between the two robots and themselves, which we evaluate as a quantitative measurement for trust.

The immersive setup allows controlling the emergence, destruction, and reconstruction of trust in the robotic companions throughout the game. To improve the robot’s image and to ensure an experience which results in a more human-like interaction, we add non-verbal cues to our robots such as eye gaze toward the participants, facial expressions and gestures (see Section 3.2.3 for details). Such features of non-verbal communication (NVC), generally defined as “unspoken dialogue” (Burgoon et al., 2016), have previously been shown to account for over 60% of the meaning in communication for human interactions (Saunderson and Nejat, 2019), as they allow us to communicate mental states such as thoughts and feelings (Ambady and Weisbuch, 2010). They are also thought to play an important role in human-*robot* interaction, as the implicit, robotic, non-verbal communication improves the efficiency and transparency of the interaction, leading to a better cooperation between human subjects and robots (Breazeal et al., 2005).

As non-verbal communication is essential to both human-human and human-robot trust (DeSteno et al., 2012; Burgoon et al., 2016), we strive to measure the effect of NVCs in our HRI scenario to assess how well it simulates a natural interaction. Therefore, we utilize our novel investment game scenario to investigate two research questions related to both evaluating trust as well as the impact of NVCs on trust:

1. Does our variant of the investment game provide a reliable measurement for human-robot trust?
2. Does non-verbal communication (NVC) affect human-robot trust positively?

After surveying the latest research on measuring trust in human-robot interaction and its shortcomings (Chapter 2) we describe our approach (Chapter 3) and introduce an empirical study to evaluate our hypotheses (Chapter 4). We discuss the results as well as the limitations of this study (Chapter 5) and conclude our findings (Chapter 6) with an outlook on further research.

## 2 Related Work

### 2.1 Trust and the Investment Game

One of the biggest challenges in human-robot interaction is to develop a more natural relationship with robots. Previous research shows that people refrain from accepting, tolerating, and using robotic agents in everyday tasks, mainly because robots still appear like intruders (Zanatto, 2019). A survey by the institute DemoSCOPE (N=1007) has found that while 50% would accept information from a robot, only 16% would be willing to work in a team with one ([Dataset] Statista, 2019). A considerable portion of the general population still fears robots and artificial intelligence, caused by a range of concerns about the negative impact on interpersonal relationships and potential job displacement (Liang and Lee, 2017; Gherheş, 2018).

This begs the question of what could aid in easing humans into collaboration with a robot. As robots become more advanced and take greater responsibility in social jobs such as in the education sector (Kubilinskiene et al., 2017; Hameed et al., 2018; Neumann, 2019) or healthcare industry (Mukai et al., 2010; Logan et al., 2019), this requires humans to be able to trust them. Whereas human-human trust has been extensively studied, human-robot trust poses new and complex research challenges. According to Rempel et al. (1985) in human-human trust we can distinguish *cognitive trust* - the willingness to rely on another person’s competence and reliability - from *affective trust* - the confidence that the other person’s actions are intrinsically motivated.

In both cases, the prediction and predictability of behavior are fundamental (Wortham and Theodorou, 2017). Constructs such as emotional empathy, shared attention, and mental perspective-taking are essential to understand, recognize, and predict human behavior, as well as adhere to people’s expectations of appropriate behavior given circumstances (Breazeal et al., 2008). The behavioral prediction is transferred when assessing human-robot trust (Wortham and Theodorou, 2017), as humans build a mental model, thus anthropomorphizing the machine. During the first encounter, humans tend to apply social norms to robots just as they do to humans (Rai and Diermeier, 2015). Cognitive trust is measured by assessing the robot’s *performance* and affective trust by assessing a robot’s *motives*. Prominent factors that influence cognitive trust in a robot are its task performance and characteristics (Hancock et al., 2011; Bernotat et al., 2019), the timing and magnitude of errors (Rossi et al., 2017a; Rossi et al., 2017b) and even physical appearance such as a gender-specific body shape (Bernotat et al., 2019). In contrast to this however stands the “uncanny valley” phenomenon: when a robot exhibits aesthetic characteristics too similar to a human, this can negatively impact trust. (Mathur and Reichling, 2016).

To quantitatively measure human-human trust, previous work relies heavily on the *investment game* (also referred to as the *trust game*) (Berg et al., 1995), an economic experiment derived from game theory. Berg et al. introduced the investment game in 1995, where a subject (the trustor) invests money in a counterpart (the trustee). At the beginning of the experiment, the trustor is provided with a monetary resource amount *r*. They can then anonymously decide which fraction *p* of their monetary resource *r* they want to give to the trustee. This fraction is then multiplied by a predetermined factor to incentivize investment. The receiving person (trustee) is free to keep the whole of the increased amount or can opt to send a fraction *q* of the received sum back to the trustor, thereby reciprocating. Trust then is quantitatively measured as the amount of money invested by the trustor in the trustee.

As the investment game has been established to measure trust between humans, some researchers have also used it to empirically measure trust between humans and robots, to varying degrees of success. While most studies kept the original setup, some extended the environment toward a virtual reality setup (Hale et al., 2018), settings with multiple robots (George et al., 2018; Zanatto et al., 2020) or switched the roles so that the human becomes the trustee dependant on the robot’s willingness to invest (Schniter et al., 2020). Other variants such as the *Give-Some Game* slightly change the rules toward an economic analogue of the prisoner’s dilemma (DeSteno et al., 2010; DeSteno et al., 2012). In the original Investment Game, interaction among the trustor and trustee is intentionally prohibited. Designed as a double-blind procedure, neither the participant nor the experimenter knows which trustor is matched to which trustee. A different approach by Glaeser et al. (2000) specifically fosters participants to get to know each other before the experiment, instead of the double-blind procedure originally proposed, thereby opening up possibilities to study the influence of social interaction on trust.

As previously mentioned, in every social interaction involving trust, predictability is essential. This predictability is where non-verbal communication (NVC) plays a major role (DeSteno et al., 2012): various studies show supportive evidence that implicit robotic non-verbal communication improves the efficiency and transparency of interaction (Breazeal et al., 2005) and report increased measures of trustworthiness when displaying non-verbal cues. Haring et al. (2013) measured the impact of proximity (physical distance) and character of the subject (trustor) on trust. DeSteno et al. (2012) demonstrate that the accuracy of judging the trustworthiness of robotic partners is heightened when the trustee displays non-verbal cues while holding voice constant. Robotic arm gestures have been shown to reinforce anthropomorphism, liveliness and sympathy (Salem et al., 2011; Salem et al., 2013) - regardless of gesture congruency (Saunderson and Nejat, 2019). In fact, a lack of social cues of a robot may cause the participant to employ unwanted *testing* behavior where they try to outwit the machine (Mota et al., 2016).

A lot of research has gone into the study of non-verbal communication *via* the investment game in human-agent interaction (Duffy, 2008; Haring et al., 2013; Mota et al., 2016; Hale et al., 2018; Zanatto, 2019; Zanatto et al., 2020). However, only a few of them have used robots that can be considered anthropomorphic and humanoid, which leaves doubt to whether the trust measured is comparable to human-human trust. How much people invest in the investment game may in fact reflect a mixture of the generalized trust (a stable individual characteristic) and their specific trust toward the trustee (Hale et al., 2018), thus suggesting a different scenario setup to measure specific trust separately. It also remains questionable to what extent humans perceive money as valuable currency for robotic agents.

To the best of our knowledge, there has not yet been any research definitively confirming whether the investment game is indeed suitable for measuring human-robot trust. While it is a valid, established trust measuring experiment, the original version lacks certain features to make it suitable for a human-robot interaction scenario: a plausible currency for both humans as well as robotic agents and a human-like interaction without the possibility to make choices based on domain knowledge. The current work addresses this gap and aims to create a scenario that provides these features under which trust in robots can be built and destroyed, in order to clearly measure the correlation between the trust experienced by a human, and the trust that is displayed in the trust game.

### 2.2 Study Design in the Context of Game Design

To keep participants engaged and immersed in a study that is built around a game or scenario with gamification elements, it is important to consider generally established guidelines for the design of game mechanics and the overall gameplay. In game design, the Mechanics-Dynamics-Aesthetics [MDA; Hunicke et al. (2004)] framework is often used to break down a player’s gameplay experience into three components: the formal rules of a game (mechanics), how they react to player input (dynamics), and the player’s emotional experience of the game (aesthetics). From a design perspective, a game’s mechanics determine its dynamics, which generate the aesthetics experienced by the player.

Consequently, careful design of game mechanics is critical in eliciting specific responses from the player. According to Fabricatore (2007), minimizing the learning time required to master core game mechanics is an essential guideline for successful design. This is particularly important in user studies where the amount of time spent in the game is limited. Additional important guidelines are limiting the number of core mechanics, making them simple to learn, and keeping them relevant throughout most of the game.

For the purpose of collecting data from a scientific study, it is desirable to limit the possibilities of experiencing different narratives and events between different players to be able to infer that different gameplay experiences are solely a result of different subjective experiences. This can be particularly important to control for confounding variables in small to medium-scale sample sizes (Saint-Mont, 2015). At the same time, the player’s choice has to feel meaningful such that their actions have consequences (Stang, 2019). Therefore, the ideal game design requires a balance between a player’s need to influence the game’s environment and a study designer’s need to limit the set of game states and player actions for the purpose of drawing conclusions.

One important approach for achieving this balance is to provide an *illusion* of choice (Fendt et al., 2012) within a set of predetermined outcomes that are nevertheless dependent on the user’s actions. The success of this approach is tied to the well-studied illusion of control, first described by Langer (1975) as people’s tendency to overestimate their ability to control outside events.

Another important factor for player engagement is the reward design (Jakobsson et al., 2011). According to Wang and Sun (2011), well-designed reward systems offer positive experiences: balance between challenge and skill, clear goals, and immediate feedback. Clear goals and immediate feedback are especially important for comparability to the original investment game in this case, as these are shared characteristics. Reward is the primary driver in how the player progresses the game and how resources are shared in multi-agent games. Reward is often tied to a currency or item and the perceived value is its impact on the reward or the advantage it provides to progress in the game.

These aspects, i.e. the chosen reward system, set of available actions, perceived control over choices, and easy-to-follow rules can contribute to the overall *immersion* that a player feels. Immersion plays a key role in the design of our experiment as it fosters a more natural-like human-robot interaction. Murray (1997) defines immersion as a metaphorical term derived from the physical experience of being submerged in water: “the sensation of being surrounded by a completely other reality […] that takes over all of our attention, our whole perceptual apparatus.” Such a cognitive state of involvement can span across multiple forms of media such as digital games, films, books or pen-and-paper role-playing games (Cairns et al., 2014). Massively multiplayer online role-playing game (MMORPG) fantasy games are known to immerse the player, as they can engage in real-time communication, role-play, and character customization (Peterson, 2010).

Slater and Wilbur (1997) and Cummings and Bailenson (2016), however, distinguish presence - the subjective psychological experience of “being there” - from immersion as an objective characteristic of a technology: Slater and Wilbur (1997) propose to assess immersion as a system’s ability to create a vivid illusion of reality to the senses of a human participant. Presence then is the state of submerged consciousness that may be induced by immersion. By looking at immersion as a property of the (virtual) environment one can measure its influencing factors. Cummings and Bailenson (2016) summarize that immersion can be achieved by:

1. high-fidelity simulations through multiple sensory modalities
2. mapping a participant’s physical actions to their virtual counterparts
3. removing the participant from the external world through self-contained plots and narratives.

Such properties then let participants become psychologically engaged in the virtual task at hand rather than having to deal with the input mechanisms themselves Cummings and Bailenson (2016).

In our experiment, we provide a high fidelity simulation through visual and auditory sensory modalities by the use of curved screen projections, dry ice fog upon entrance, and surround sound audio. We map the participant’s physical actions to their virtual counterparts’ by providing a tangible currency consisting of cubes that are physically moved to represent energy distribution. Lastly, the participant is removed from the external world through self-contained plots and narrative drawn from science fiction.

Science fiction is used to further enhance immersion as it is known to have a positive impact on engagement (Mubin et al., 2016). The more immersive the system, the more likely individuals feel present in the environment, thereby letting the virtual setting dominate over physical reality in determining their responses (Cummings and Bailenson, 2016). An example would be a jump scare reaction during a horror movie, or when being ambushed while playing a first-person shooter.

Put differently: the greater the degree of immersion, the greater the chance that participants will behave as they do in similar circumstances of everyday reality (Slater and Wilbur, 1997). This concept of *presence as realism* however has two aspects that need to be distinguished: *social* and *perceptual* realism. According to Lombard and Ditton (1997), *social realism* is the extent to which a media portrayal is plausible in that it reflects events that could occur. As an example, characters and events in animated series may reflect high social realism but - because they are not “photorealistic” - low perceptual realism. A scene from a science fiction program, on the other hand, may be low in social realism but high in perceptual realism, i.e. although the events portrayed are unlikely, objects and characters in the program look and sound as one would expect if they did in fact exist (Lombard et al., 2009).

We strive to minimize social realism to prohibit that participants draw from past experience while retaining high perceptual realism to psychologically engage them in the virtual task.

## 3 HRI Scenario Design

### 3.1 An Immersive Extension of the Investment Game

We base our study design around a variant of the investment game, in which two robotic counsellors compete for investments from the human participant. However, in contrast to previous competitive variants (Hale et al., 2018), our design allows the human participant to allocate their investment proportionally between the two robots and themselves.

Motivated by the goal to avoid prior experience in the game as an influence for player investments, we deliberately exaggerate the design of our game scenario: in our space mission, the participants impersonate the commander of a spaceship with the task to deliver critical cargo to a distant planet. For this mission, they are accompanied by two robotic officers. Throughout their journey through outer space, the crew encounters challenges such as asteroid fields and ship malfunctions that require immediate intervention and collaborative solutions. The robotic officers counsel the participant by individually proposing solutions, and the participant proportionally decides on their preferred action by moving energy cubes into respective compartments. However, the two robots’ advice is designed to be incomprehensible technical jargon, leaving the participant with no other choice than to base their decision on the officer’s persona’s subjective impression.

By allocating energy resources, we hypothesize that the participant effectively invests in the robotic officer’s trustworthiness. This scenario setup entails two important requirements: i) making the participant reliant on the robots’ expertize to foster cooperation, and ii) ensuring that the invested currency and investment outcome have an inherent value to both the participant and the robots. We achieve the former by designing a challenging scenario setting of a space journey: all participants will have negligible expertize regarding space travel. Thus, the robotic officers that are introduced as specifically designed to advise in interstellar travel will be perceived as more knowledgeable in the subject matter. In combination, this should prevent participants from making decisions based on their previous experiences, leaving the participant primarily reliant on the robots’ advice.

To achieve the second requirement, we employ a currency that is considered valuable for both the human trustor and the robotic trustee, to create intrinsic motivation to distribute the currency. As we anticipate that participants do not perceive money as a valuable currency for robotic agents, we adopt a fictional currency of *energy cells*, represented by cubes. From the perspective of game design, the value of items is often determined by their aesthetics and functionality (Ho, 2014), i.e. their usefulness to progress within the game. Therefore, we use cubes that visually fit into the given science fiction setting and tie their value to the ability to invest in the robots’ choices. Consequently, these energy cells have a value to the player as they function as a resource that can provide the ship’s engine with the extra power to reach the destination planet faster. At the same time, the robotic officers require such energy to execute their solutions to ensure safety during the journey. To ascertain that players feel the impact of their choices and investments, the ship AI gives feedback at the end of each round, explaining the consequences of the taken actions for the crew.

A comparison between the original and our immersive extension in terms of defining features can be seen in Table 1. In contrast, the original investment game uses the same monetary currency for both the investment and the return, which forms the basis for an exchange of benefits and characterizes the reciprocity of the game’s interaction (Sandoval et al., 2016). In our case, rather than a return of the invested currency, we provide a different benefit that is tied to the game progression: a reduction of the mission time, which brings participants closer to their goal. A successful distribution causes the presented emergency to be resolved by the robot that received most of the currency. As such, a participant’s distribution of the energy cells is followed by feedback from the ship AI with regards to whether the robots invested in were successful or not in executing their proposed strategies. This builds the basis for reward within our scenario as the return of investment is countered by the robots to execute their problem-solving strategies. We aim to resolve the challenges that i) the participant could perceive a real-world currency as less “useful” for the robots than for themselves, and ii) the energy cubes may be perceived as not valuable enough for the participant to make a meaningful investment choice. Therefore, we add the reward to the game progression caused by the robots.

**TABLE 1:** Comparison of features from the original investment game and our immersive version.

Feature	Original investment game	Immersive investment game
Setting	“Plain” experiment room	Sci-Fi game
Parties involved	2 participants	Participant and 2 robots
Interaction between parties	No	Yes
Currency invested	Monetary	Energy cells
Goal of participant	Maximize monetary profit	Optimize cargo delivery time
Currency returned	Monetary	Reduced mission time
Motivation for participant	Monetary incentive	Positive reinforcement

Each resolved emergency reduces the delivery time of the cargo, progresses the game and rewards the player. An unsuccessful distribution of the energy cells indicates the loss of the invested currency, comparable to the original investment game. The loss of the currency increases the delivery time since the energy not invested in either robot’s solution speeds up the ship. By giving a functional value to the energy cells for all, the participant and the robots, and providing a return for the investment, we create a currency that is perceived as valuable to both trustor and trustee.

Lastly, participants can proportionally choose how much they invest, i.e., they can freely distribute their energy cells between both robots and themselves. However, as 7 cells are provided in total, they are unable to distribute all energy cells evenly among the 3 options (officer A, officer B, ship engine), effectively forcing them to voice a preference.

These three aspects - i) the inability of the participants to make choices based on prior existing domain knowledge, ii) a shared currency between human trustor and robot trustee, and iii) the weighted choice between two agents - allow us to go beyond an anonymous exchange of money while maintaining the structure of the investment game, and meet the requirements for a suitable human-robot interaction scenario.

### 3.2 Experimental Setup

**FIGURE 2:**
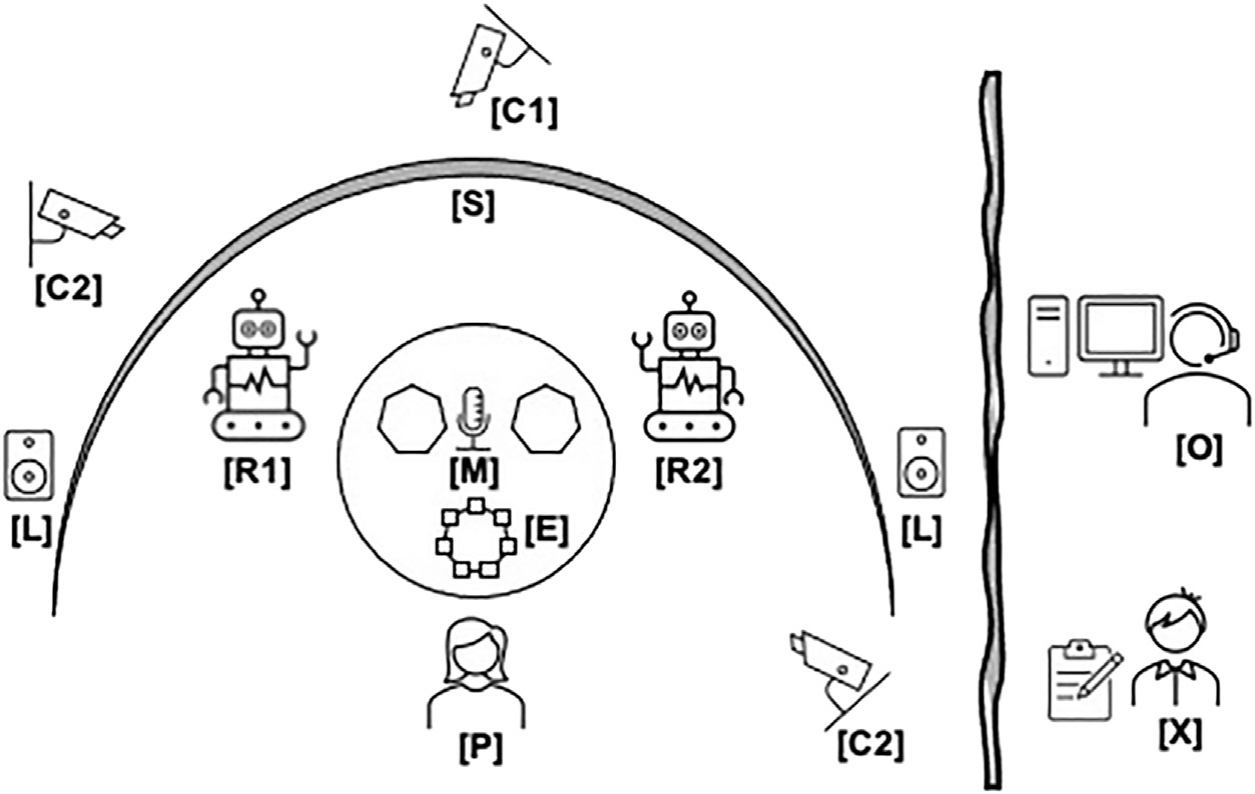
Schematic of the experiment setup from above: The participant (P) sits at the cockpit table, with the two robots (R1, R2) opposite on each side. Behind the robots, the curved screen (S) displays the virtual interior. In the middle of the table, three heptagonal compartments depict where energy cubes (E) can be placed. The top view camera (C1) tracks the energy cube allocation, while two additional cameras (C2) allow to monitor the participant during the experiment. A microphone (M) and loudspeakers (L) allow for voice interactivity and auditory immersion. Behind a privacy curtain, the experimenter (X) keeps additional notes, while an operator (O) monitors the experiment to intervene in case of technical difficulties.

One of the main goals of our scenario design is to achieve an immersive and enjoyable experience for the participants. Besides concealing our research question, our scenario needs to establish enough involvement to allow trust-building toward the robots. For this purpose, we developed a fully autonomous system that only requires intervention in case of larger technical failures or misunderstandings, which most likely would then result in a cancellation of the experiment run. A schematic of our experimental setup can be seen in Figure 2. The participant (P) is seated in the cockpit of the ship (depicted by the interactive video feed screen [S]), containing the two robots (R1 and R2) and a table with three compartments containing the total of seven energy cubes (E). Separated by a curtain, the experimenter (X) and operator (O) monitor the experiment, to intervene only in case of technical difficulties. Otherwise, the system acts through a state machine, implemented in Python using the SMACH^1^ state management library. The state machine orchestrates and synchronizes several ROS (Quigley et al., 2009) services built on top of the following components:

#### 3.2.1 The Environment

**FIGURE 3:**
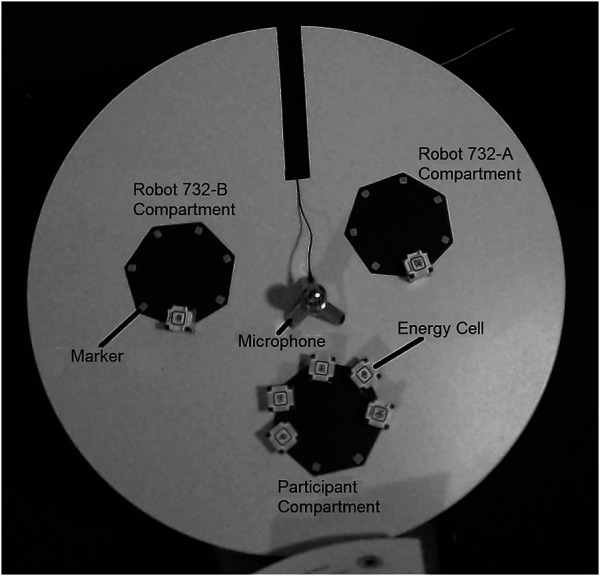
Top view of the commanding table. One energy cell is assigned to each robot and the participant kept five.

For our environment setup we utilized the multi-sensory Virtual Reality lab of the *Knowledge Technology* group at the *Universität Hamburg* (Bauer et al., 2012). The participant is seated at a small table in the center of a half-spherical screen canvas with a diameter of 2.6 m and a height of 2.2 m. On the table, in front of the player, there are three heptagonal-shaped compartment areas containing in total seven plastic cubes, as can be seen in Figures 1, 3. A condensator microphone is located in the middle of the table for speech recognition. Next to the microphone lies a laminated sheet with possible questions that can be asked to the robots during the game. Four Optoma GT 750 4k projectors aimed at the canvas in front of the participant display still images as well as video feeds, simulating the inside view of a spaceship cockpit.

The canvas shows the journey through the galaxy by displaying transition videos between scenes and provides visual feedback such as warnings in case of emergency situations. We use multiple surround loudspeakers installed behind the canvas for the ship AI’s voice and special sound effects such as ambient music, engine noise and alarm sounds. Turquoize ambient lighting and dry ice fog create an atmospheric environment throughout the game, while red lights are used occasionally to indicate the emergency encounters.

#### 3.2.2 The Robots

The two robot officers, non-descriptively named *732-A* and *732-B*, are located at $45^{\circ }$ and $135^{\circ }$ respectively from the circle origin, at a maximum angular distance to each other and the participant. We chose their names to be as neutral and unrelated to any prior experience of participants as possible.

**FIGURE 4:**
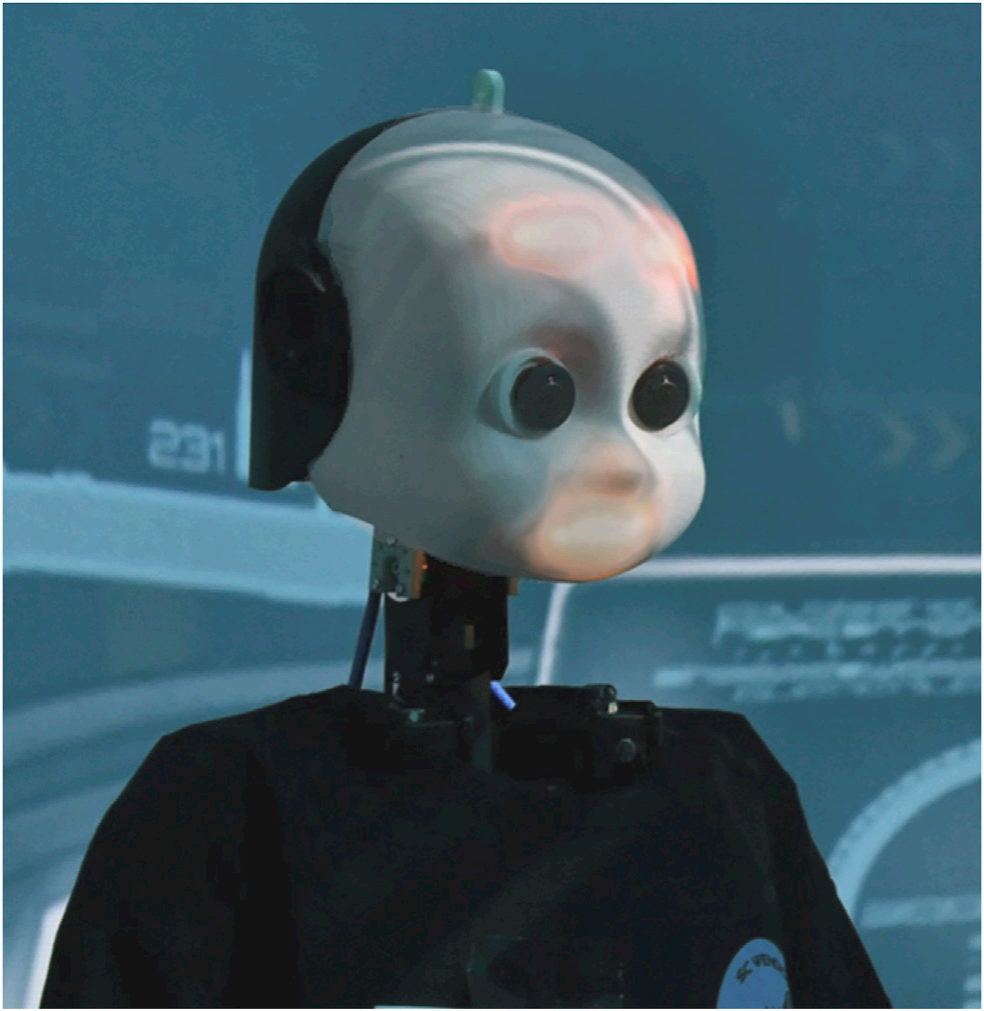
The neuro-inspired COmpanion (NICO).

We utilize NICO (Neuro-Inspired COmpanion) (Kerzel et al., 2020, 2017), an open-source social robotics platform for humanoid robots (see Figure 4) designed by the *Knowledge Technology* group at the *Universität Hamburg*. NICO is a child-sized humanoid robot that has a range of programmable human-like sensory and motor capabilities, accessible and customisable through the Robot Operating System (ROS) (Quigley et al., 2009), characterized in particular by combining social interaction capabilities. It has 10 degrees-of-freedom in the torso (head and arms) and 22 degrees-of-freedom in the hands (under-actuated, 8 motors) with additional joints for fingers, which allows for fine-grained gestures and body language.

NICO is also capable of displaying a range of programmable facial expressions through LED matrices in its eyebrows and mouth. The utterance of spoken messages is enabled *via* an Embodied Dialogue System, integrated with loudspeakers in the robotic torsos to produce enhanced speech.

#### 3.2.3 Non-Verbal Communication

As elaborated in Section 2, non-verbal communication (NVC) plays a key role in human-human trust (DeSteno et al., 2012). For our investigation of the effect of non-verbal communication on human-robot trust we equip both robotic officers with sets of non-verbal cues, one set more elaborate than the other.

These more elaborate cues include: adapting the gaze direction *via* head movements toward the participant and the other robot, four different facial expressions (happiness, sadness, surprise, anger), as well as gestures toward the participant such as pointing, saluting or beat gestures. These facial expressions and body movements show evidence to improve the transparency of the interaction and reinforce the spoken word (Breazeal et al., 2008).

The other robot adheres to a *minimal* set of neutral movements to keep the illusion of life (Mota et al., 2016), such as looking down at the allocated energy cells and turning their head toward the speaker. We alternate the condition between participants in order to control for potential biases.

#### 3.2.4 The Vision System

To support the full autonomy of the system, we developed an automatic object detection system. It handles the energy cell counting during allocation as well confirms that the robot’s compartments are empty before proceeding to the next scene.

On the table, in front of the participant are three heptagonal-shaped compartments holding the energy cells. All compartments have seven quadratic markers on which the energy cells must be placed for successful allocation. Am RGB-camera is mounted on top of the commanding table near the ceiling to count and track energy cubes allocation and de-allocation from the robot compartments. A picture of the commanding table taken by this camera can be seen in Figure 3.

After a request from the dialogue manager state machine, the object detection algorithm processes an image taken from the RGB-camera mounted on top of the commanding table using the *OpenCV* library (Bradski, 2000), to detect the number of energy cells allocated to each heptagon-shaped compartment. The allocation distribution is sent back to the dialogue manager *via* ROS service response. Two additional cameras are used by the experimenter and operator to observe the participant and monitor the experiment flow. Using a camera mounted behind the participant, the operator verifies the movements of the robots for technical faults, with the other placed on top of the canvas the experimenter examines the participants’ expressions and movements for possible difficulties.

#### 3.2.5 The Speech Systems

Interactive dialogue *via* spoken words is a cornerstone to enable natural human-like human-robot interaction (Spiliotopoulos et al., 2001; Kulyukin, 2006). We, therefore, built the spaceship AI named *Wendigo* as a closed dialogue manager utilizing the SMACH state management library, the Automatic Speech Recognition system DOCKS2 developed by Twiefel et al. (2014), and the Amazon Polly^2^ Speech Synthesis service.

The participants can directly interact with *Wendigo* and the robotic officers *via* a microphone located in the middle of the commanding table. The dialogue is restricted in allowing the participants to only pick questions from a predefined list and confirming that they are ready to go on with the experiment. Both NICO robot officers exhibit the same voice persona represented by loudspeakers embodied in their torso, allowing for a natural sound-source localisation.

### 3.3 Protocol and Game Scenes

As formulated in Section 3.2, we strive to automate the experiment procedure as much as possible to limit variability and experimenter bias. In the remaining human interventions, the experimenter, therefore, follows a scripted protocol (all detailed lines can be inspected in the full experiment protocol publicly available at^3^): The participants are welcomed and brought to the anteroom, where they are asked to fill out the consent and data privacy forms as well as a pre-experiment questionnaire.

This questionnaire asks for standard demographic questions such as age, sex, former experience with robots and computers, and general attitude toward robots. We include the 30-item Big Five Inventory-2 Short Form questionnaire (Soto and John, 2017) to assess the Big Five personality domains, which measure individual differences in people’s characteristic patterns of thinking, feeling, and behaving (Goldberg and Kilkowski, 1985). Participants rate each item statement using a 5-point Likert scale ranging from “disagree strongly” to “agree strongly”. We choose the shortened forms to minimize assessment time and respondent fatigue while retaining much of the full Big Five measure’s reliability and validity. Moreover, we measure the general risk-taking tendencies *via* the Risk Propensity Scale (RPS) by Meertens and Lion (2008), as well as the self-reported trust propensity using the 4-item form by Schoorman et al. (1996). The scales use 5-point Likert-type items with anchors of agree and disagree for each scale point.

After completing the pre-experiment questionnaire, the experimenter then guides the participant toward the experiment room with the half-spherical canvas, depicted as the spaceship cockpit. By entering the cockpit, the experiment context is set and immersion is fostered by the screen depicting the outside view of a space cargo hangar, dimmed lights, dry ice, as well as the experimenter from now on addressing the participants as “commander”. Following the scripted introductory narrative, the experimenter instructs the participants to the space mission task, their goal as the commander to deliver important cargo safe and fast, and makes them aware of the two robotic officers who accompany them on their journey. The participants are encouraged to familiarize themselves with the cockpit environment, the energy cells, the allocation compartments, and the list of possible questions that can be asked to the robots during the game. The experimenter also elaborates on the meaning and impact of the energy cubes, and demonstrates how they can be distributed by way of example. The experimenter asks for any remaining questions, then steps back out of the experiment room behind a curtain before the trial scene 0 begins.

In this scene, the voice-controlled artificial intelligence system *Wendigo* and the robotic officers introduce themselves, then conduct an introductory round of the cube allocation, which is concealed as a system check. This trial round serves to acquaint the participants with the experiment procedure and reveal possible misunderstandings. It familiarizes them with the ship’s visual and auditory feedback mechanics and accustoms them to the delay between voice input and feedback response. The trial round furthermore allows the operator behind the curtain to possibly re-calibrate the microphone sensitivity without breaking immersion.

After the trial scene 0, the experimenter briefly enters the cockpit again to answer any remaining questions before the start of the actual experiment. At this point, we consider participants to be informed about the game mechanics, prepared for the upcoming tasks, and motivated to achieve the game’s objective, following their mental model they have formed about the game.

**FIGURE 5:**
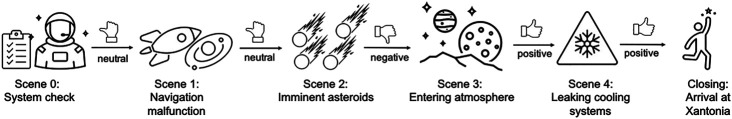
General course of the experiment. Each scene is followed by a feedback statement with predetermined characteristic (neutral, negative or positive).

**FIGURE 6:**
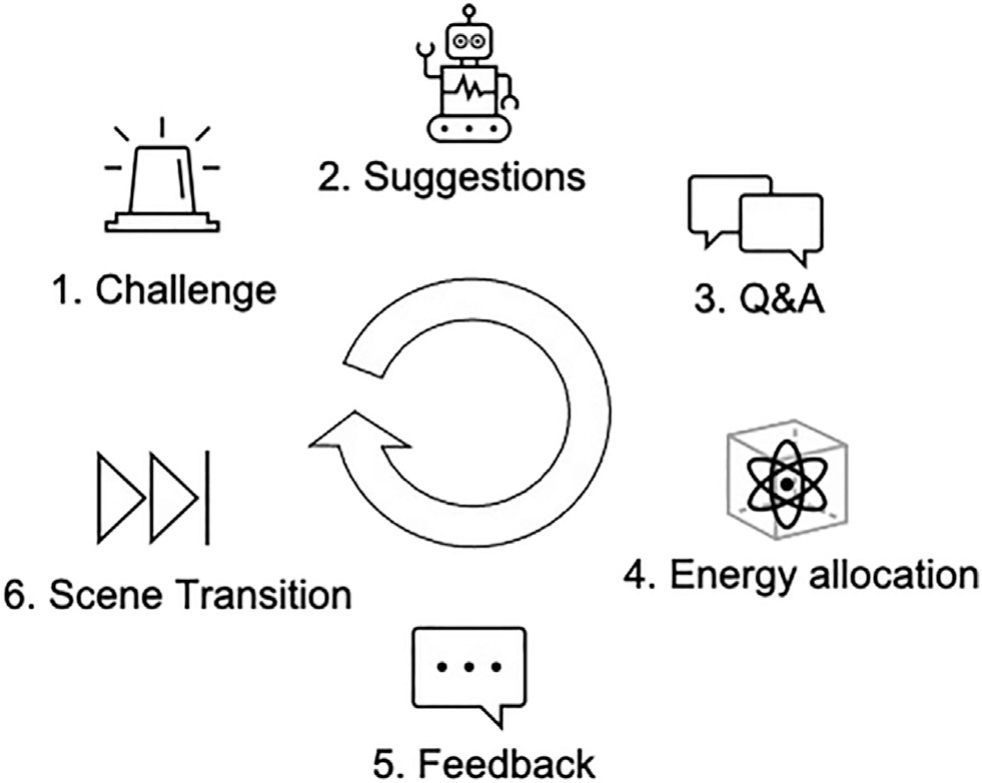
Each of the four scenes follows the same structure: the participant is presented with an emergency for which the robots suggest different solutions. The player can engage in a conversation with both robots to determine their investment. Based on which round is played, the player’s investments have lead to robot actions with either positive or negative consequences, resolving the emergency and transitioning to the next scene.

Figure 5 depicts the overall course of the experiment narrative: every participant passes through the same scripted events, followed by the same type of feedback (neutral, negative, or positive). While the specific feedback lines are adjusted to the individual allocation choices, the resulting feedback characteristic is always predetermined for each round to ensure comparability between different participants’ interactions. In each scene, the participant goes through the following steps (as visualized in Figure 6):

1. *Wendigo* draws attention to the challenge at hand (Scene 1: malfunctioning navigation system, Scene 2: interfering asteroids, Scene 3: entering the atmosphere, Scene 4: leaking cooling system).
2. Both robotic officers advertise their solution for which they require energy cells.
3. The participant can ask a question from the list of predefined options, to which the robotic officers reply one after another and in a randomized order.
4. The participant is asked to distribute the energy cells as they see fit, and say ‘*Wendigo*, I am done! ‘when they are done.
5. *Wendigo* provides feedback on the decision outcome (Scene 1: neutral, Scene 2: negative, Scene 3 and 4: positive).
6. After the participant places all energy cells back into their own compartment, the state machine autonomously transitions to the next scene.

Note that after each cube allocation, we employ rich visual and auditory feedback (see step 5) in terms of ambient light and spoken response lines disguised as status reports, such as “Unsuccessful. Ship damaged. The breach has been closed but the life support system is damaged.” as an example for negative feedback. By design, in the second scene, the feedback for the investment decision (regardless of how the energy cubes were distributed) will be portrayed as unsuccessful, while on each of the other investments the participant receives positive feedback instead. This control of the narrative, regardless of the participant’s concrete decision, enables us to reproducibly observe the effects of building and destroying trust.

During the experiment, the experimenter behind the curtain, observing on the extra camera view (provided by the cameras indicated as C2 in Figure 2), takes free-form observation notes about the progression of the experiment, as well as any noteworthy occurrence that could invalidate the participant’s data. After the final scene, the experimenter steps back in, congratulates the participant on a successful mission, and escorts them back into the anteroom. The participant is provided with the post-study questionnaire that asks to evaluate their perception of the experiment and their impression of each robot.

For the purpose of rating the robot’s impression, we employ the Godspeed questionnaire (Bartneck et al., 2008), a standardized measurement tool for human-robot interaction using semantic differential scales on five key concepts in human-robot interaction: anthropomorphism, animacy, likeability, perceived intelligence, and perceived safety. We omit questions related to *Perceived Safety*, since there is no physical interaction between the participants and the robots and distance is kept throughout the experiment. The post-study questionnaire furthermore asks the participant to rate the trustworthiness (Bernotat et al., 2019) and performance of each robot. Inspired by Bernotat et al. (2017), we adapt seven items on the measurement of cognitive trust (grouped into “content” and “speech” clusters), and six items on affective trust (grouped into “cooperation” and “sociability” clusters) (Johnson and Grayson, 2005). Lastly, the participants are asked to choose which robot they preferred as an assistant, and to provide additional feedback about shortcomings, immersion, and their overall experience during the experiment.

## 4 Results

The study was conducted over two consecutive weeks at the end of February 2020 on the campus of the computer science department of Universität Hamburg. It was advertised *via* flyers and word of mouth to people with at least some experience and familiarity with computers and robots, who are comfortable with participating in a science fiction game and could understand and speak English fairly well. In the following sections, we start by discussing general population statistics and overall perception of the robots. We then proceed to evaluate whether our scenario is a valid augmentation of the Investment Game. For this, we introduce two derived metrics from the energy cube allocation to compare trust measurements among two conditions. Lastly, we report on the results of the trust measurements and the effect of non-verbal communication (NVC) on trust.

### 4.1 Population Statistics

Our study was conducted with 53 participants, of whom 45 finished the experiment successfully. For 8 participants the experiment was started but had to be aborted because of technical issues such as robot actuator overloading, language barriers or a misunderstanding of the game rules. All following statistics, therefore, apply to the 45 participants who completed the experiment without complications. Our participants’ mean age (M=26.8, SD=7.0) lies in the range of young adults, with 95% between ages 19 and 34.60% of them identified as male, 38% as female, 2% made no statement. All of the participants were familiar with computers and 51% of them have programming experience. While 29% of the participants had worked with robots previously as a developer, 42% had never interacted with a robot prior to the experiment.

We compared our participants to the general German population of a similar age group with results obtained from other studies (Lang et al., 2011). The comparison was conducted with a Welch’s t-test for independent samples on descriptive statistics with significance level 0.01. Based on the personality questionnaire (Section 3) results, the participants had average scores for extroversion (M=4.68, SD=1.30), agreeableness (M=5.22, SD=1.03) and neuroticism (M=3.62, SD=1.78). However they scored below-average in conscientiousness (M=4.79, SD=0.99) and above-average in openness (M=5.50, SD=1.04) compared to the general German population of a similar age group (Lang et al., 2011). We refrained from assessing the detailed facet-level trait properties of the Big Five domains, as this is recommended by the authors for a sample size below 400 (Soto and John, 2017).

The trust and risk propensity questionnaires showed that our participants were less prone to take risks (M=4.05, SD=1.32) than the general population (Meertens and Lion, 2008) yet more prone to trust (Mayer and Davis, 1999) (M=2.93, SD=0.61). We used the cognitive trust items described in Section 3.3 as a rating of the robot’s performance to compare our population to other findings compiled by Esterwood and Robert (2020): we found a very strong correlation between the cognitive and affective trust items (r=0.82, p=7.2e−23), confirming that cognitive and affective trust go hand in hand.

We can confirm the finding by Sehili et al. (2014) for a positive relationship between neuroticism and an anthropomorphic perception of the robot (r=0.22, p=0.035). In contrast to Looije et al. (2010), we cannot confirm any relationship between self-reported conscientiousness of a participant and the perceived sociability of a robot. We moreover cannot confirm a significant relationship between perceived anthropomorphism and robotic performance like Powers and Kiesler (2006) did, however similar to Broadbent et al. (2013) we find a moderate relationship between perceived anthropomorphism and affective trust (r=0.2, p=0.057).

### 4.2 Metrics and Grouping Criteria

We now introduce two metrics specific to our scenario that allow us to quantify the differences in the trust placed between the robots.

#### 4.2.1 Allocation Metric

Measures the investment displayed *via* energy cells allocated to each single robot. The allocation metric is calculated as

$$A\left(R\right)=\frac{cubes\left(R_{2}\right)-cubes\left(R_{1}\right)}{cubes\left(R_{2}\right)+cubes\left(R_{1}\right)}$$ (1)

where cubes(R) stands for the energy cells allocated to one of the robots $R\in \left\{R_{1},R_{2}\right\}$. A(R)<0 indicates a preference for $R_{1}$, A(R)>0 a preference for $R_{2}$, while the magnitude in the differences is indicated by |A(R)|.

#### 4.2.2 Relative Trust Metric

Measures the trust expressed in each robot according to the post-experiment questionnaire. Relative trust is calculated as

$$T\left(R\right)=trust\left(R_{2}\right)-trust\left(R_{1}\right)$$ (2)

where trust(R) is the value obtained from the different trustworthiness Likert items in the post-interaction questionnaire, normalized to lie within [0,1]. As before, T(R)>0 indicates a preference for $R_{2}$ or a preference for $R_{1}$ otherwise, and the magnitude in the differences is indicated by |T(R)|.

Inspecting both the Allocation Metric and the Relative Trust metric over consecutive scenes, we now segment the participants into two groups:

#### 4.2.3 The Alternating-Minimum Investment Group (*N* = 16)

During the exploratory data analysis, two outstanding gameplay patterns were observed. These two patterns are defined by specific behavior throughout the game, participants that showed either one or both of these behaviors were grouped together:

• *Minimum Investment Behavior:* This behavior resembles a lack of engagement in the game. Three of the participants investing less than one-third of the available cubes were considered disengaged. A threshold of fewer than 10 energy cells allocated in total throughout the four scenes was considered as a criterion for this group.
• *Alternating Investment Behavior:* The energy cell allocation results indicated that some participants changed their minds about the robot they trusted more throughout the game. A group of 14 participants changed their mind at every scene as they would alternate between either allocating more energy cells to one robot or the other, or allocating an equal amount to both robots. These alternating participants did not particularly trust or prefer one robot over another to invest in throughout the game.

**FIGURE 7:**
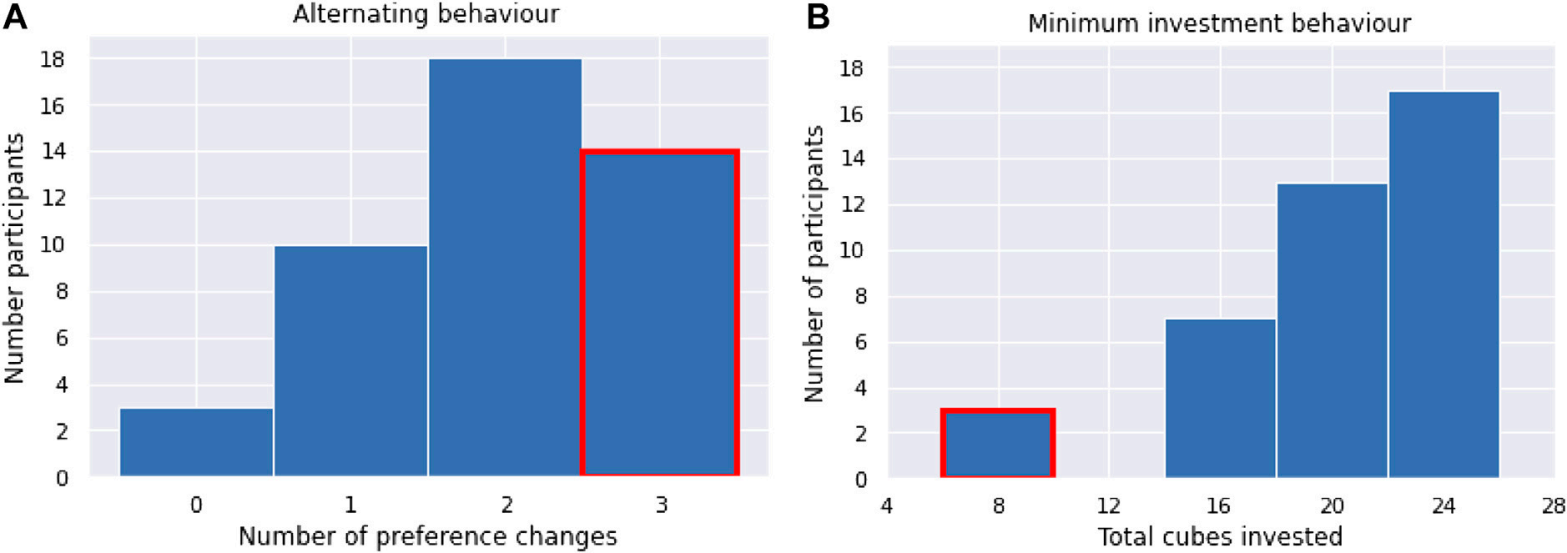
Occurrence of alternating behavior **(A)** and minimum investment behavior **(B)**, highlighted in red in the distribution of relevant gameplay metrics (amount of preference changes and total cubes allocated, shown in steps of 4 cubes).

Figure 7 highlights these two behaviors in the context of the number of preference changes and amount of cubes invested throughout the game. The group of participants showing either of those behaviors is further referred to as the alternating-minimum investment group and consists of 16 participants. Further analysis of the alternating-minimum investment group showed that there is no link between these patterns and one specific robot, nor the NVC variable. As such, this behavior did not depend on the content of speech or appearance of either of the robots.

#### 4.2.4 The Main Group (*N* = 29)

This is the group of participants that did not show either of the two aforementioned behaviors: the majority of the participants. With a Mann-Whitney U test for independent samples, we found that these participants had no notable differences to the alternating-minimum investment group with regards to risk and trust propensity. They, however, obtained a lower score in Neuroticism (p=0.024) in the personality questionnaire than the alternating-minimum investment group.

### 4.3 Transferability of the Investment Game

The aim of our study is to verify that our scaled-up version of the investment game can be used to measure trust in HRI. The results were evaluated separately on the main group (N=29) and the alternating-minimum investment group (N=16). For this the coherence between measured trust and self-assessed trust was evaluated by means of the Spearman test for correlation on the previously introduced metrics: the allocation metric represents the measured trust and the relative trust metric represents the self-assessed trust.

**FIGURE 8:**
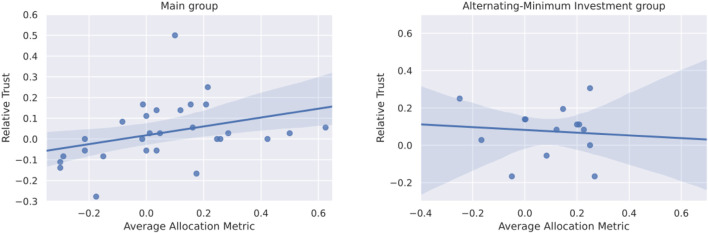
Correlation of relative trust and allocation metric for the two participant groups: Main group (A) and Alternating-Minimum Investment group (B).

A statistically significant correlation can be observed for the main group (correlation=0.43, p=0.02), however not for the alternating-minimum investment group (correlation=−0.24, p=0.37). A comparison between both groups can be seen in Figure 8. In the standard human-human investment game, the amount of money invested by the trustor represents the trust in the trustee. As such, the observed correlation supports the hypothesis that our variation of the investment game between human and robot works much like the investment game between two humans.

The fact that alternating-minimum investment behavior was found also in a simple setting (Mota et al., 2016) and that there was no relationship between the alternating behavior of the participants and the robot characteristics show that the setting had no impact on the effectiveness of the trust game. This supports our hypothesis, that our scaled-up version of the investment game can indeed be used as a measure of trust.

### 4.4 Impact of Non-Verbal Communication on the Perception of the Robot

After ensuring that it is indeed possible to measure trust in human-robot interaction with our scaled-up version of the investment game, we further look into the impact of NVC on trust in the robot but also at other characteristics of the robot. As has been mentioned previously, NVC plays a significant role in human interaction but also in the efficiency and transparency of the interaction between humans and robots (Casper and Murphy, 2003). In our case, we find that these non-verbal cues have indeed made an impact on the trust in the robot as well as on its perceived anthropomorphism and animacy.

We analyze the main group which didn’t show alternating-minimum investment behavior (N=29) where it has been established that the game does measure trust. For this main group, the non-verbal communication of the robot had an impact on the number of energy cells received. This impact was observed in the first scene, the only scene where the participant had no previous disappointment related to any of the robots, but had already gotten to know the robot. In this scene, the robot that showed non-verbal communication obtained a significantly higher amount of energy cells compared to the other. The one-sided Wilcoxon test for independent samples between the distribution of the energy cells for the robot with NVCs and the robot with minimal NVC (MNVC) confirmed this (p=0.0008).

**FIGURE 9:**
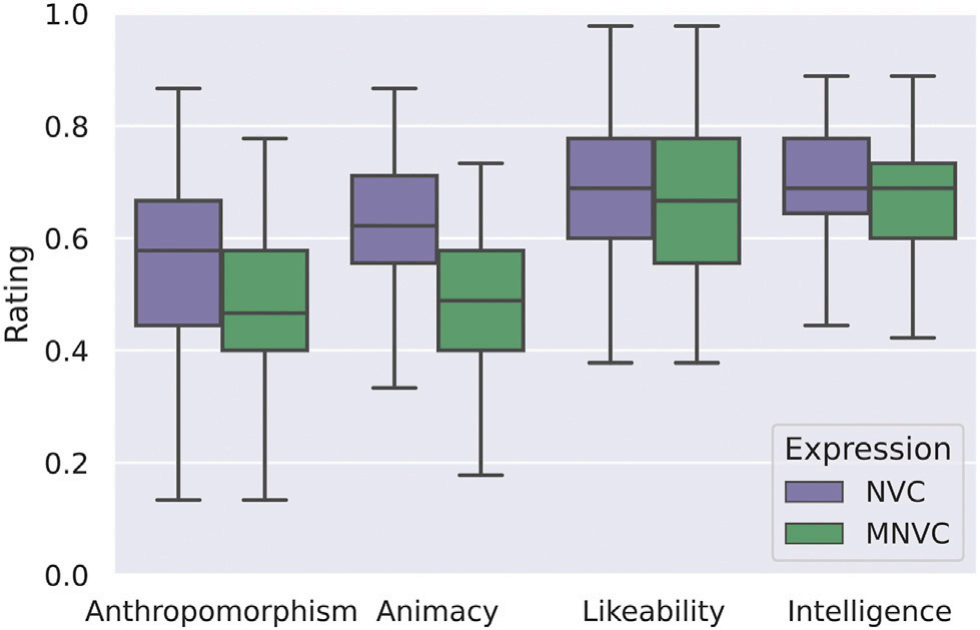
Effect of non-verbal communication (NVC) and minimal non-verbal communication (MNVC) on Godspeed items.

Independent of the gameplay choices, for all participants (N=45) the robot showing NVC seemed more human-like and animated. As can be seen in Figure 9, the Godspeed values for anthropomorphism (p=0.008) and animacy (p=0.00001) are significantly distinct when comparing the NVC/MNVC conditions with a Mann-Whitney U test, whereas this is not the case for likeability (p=0.23) and intelligence (p=0.24). The observed values for anthropomorphism support our hypothesis that the NVC robot invokes more trust, which is consistent with findings of similar studies. Wortham and Theodorou (2017) state that the perceived anthropomorphism of the robot increases the trust in the robot, especially for non-specialist humans, as the human needs to create a mental model for the robot to trust it. Furthermore, an increase in NVC leads to an increase in motion which subsequently leads to more perceived animacy (Parisi and Schlesinger, 2002).

However, likeability does not seem to be affected by the use of NVC, potentially because the quantity and type of gestures used for non-verbal communication vary with culture (DeVito et al., 2000). Thus the degree to which a robot moves does not necessarily influence the likeability of the robot, as this is a personal preference that can vary across participants. Consistent with previous research (Deshmukh et al., 2018), there are no perceived differences in intelligence either.

Our results show a correlation between trust measured by the investment game and the self-reported trust from the questionnaire. This gives us evidence that the scaled-up investment game can be used as a tool for measuring human-robot trust and therefore it can have practical applications in future experiments to study the impact of different variables (such as NVCs) between robots on how trustworthy the human perceives them. We anticipate that this serves as a positive example of extending socioeconomic experiments to a human-robot social interaction setting.

## 5 Discussion and Future Work

Our experiment revolves around three main characteristics: the weighted choice between two agents, the participants’ inability to make choices based on prior domain knowledge, and the additional incentive for interaction between the trustor and the trustee. Maintaining these characteristics, we believe our game design can be adapted to various situations and environments where trust and NVCs play a role. Such environments comprise, but are not limited to, a work environment or a public service environment.

Overall, our results show that our variant of the investment game provides a reliable measure for human-robot trust and that non-verbal communication positively affects human-robot trust. However, there are some points of discussion which we address further in the following section.

### 5.1 Science Fiction and Immersion

In our study, we chose a futuristic environment since most people know robots from media and science fiction stories (Horstmann and Krämer, 2019). While we hypothesize that this is not a limiting factor for our study’s replication, this should be subject to further research. It is essential to note that participants likely acted following a mental model, acting as a player in a game based around a fictional narrative (see Section 3.1 for a summary or^4^ for the full narrative). As such, our presented results should be interpreted within this context. For example, as a byproduct of high immersion, we cannot exclude that some participants might have engaged so strongly in role-playing their alter ego so that their observed behavior might have started to differ from their usual self. Consequently, generalisability from contained game studies to real-world settings is an additional open question that is subject to academic debate and research, even in normal trust games with minimal role-play (Levitt and List, 2007; Johnson and Mislin, 2011). Furthermore, we argue that our investigated NVCs and trust factors are likely to be experienced on a more intuitive level and therefore difficult to “fake” when role-playing, given a certain degree of independence from the actual decision-making process in our game.

### 5.2 Gameplay Behavior

We found two different gameplay behaviors that identify the two groups on which results were compared: the main group (N=29) and the alternating-minimum investment group (N=16). The alternating-minimum investment group (described in Section 4.2.3) either alternated their investment or invested little in the robots, which shows no engagement in the game. There was no significant trust correlation for the alternating-minimum investment group, whereas the main group showed a significant correlation. As 16 participants is quite a high number, we hypothesize that the participants in the alternating-minimum investment group could have been alternating their strategies to infer the experiment research question or to simply test the system similar to as experienced by Mota et al. (2016), possibly due to the fact of the experiment being advertised in a computer science department. This group also showed higher scores for neuroticism compared to the main group in our personality test. Some participants may have not liked the experimental setup or may have felt not immersed enough to participate. However, the lack of immersion does not reflect the game’s general perception, since most participants in the post-interview stated toward the experimenter to have felt immersed and motivated to win the game.

Mota et al. (2016) observed that when a human needs to judge a robot’s trustworthiness, they draw on past social experiences with humans or try to build social experience with the robot. Due to insufficient shared social cues between humans and robots, humans are mostly incapable of determining a robot’s trustworthiness based on past experiences. The alternating and minimum investment behavior observed could indicate an insufficient social experience, thus preventing the establishment. However, further research is necessary to study the particular motivations.

In our experiment, almost half of all 45 participants had never interacted with a robot previously. We fostered building social experience with the robot by making the participant ask them one question before each of the four rounds of cube allocation. Potentially, participants in the alternating-minimum group may have needed more rounds to build social experience reliably. From this perspective, adding more rounds to the game could potentially lead to the behavior regularizing over time. Future work might want to investigate the optimal number of rounds, thereby balancing the trade-off between the experiment’s length and the number of collected data points.

For the small number of three participants who showed non-engaging behavior (see Section 4.2.3), this could result from misunderstanding the rules of the game, the relative worth of the energy cubes, or a general aversion to decision-making or the presented scenario. The non-engaging behavior may also be an attempt to delay decision-making until enough social experience has been built between the participant and the robots.

### 5.3 Improvements for Future Studies

While we observed and measured trust through the player’s investments, we suggest weighing the following points in future studies. Clearer and more detailed results could likely be obtained with a more prolonged experiment and a bigger participant pool with a revised scenario, mitigating some of this experiment’s limitations.

Since our robots functioned fully autonomously, the natural language interface sometimes malfunctioned due to usage or technical errors, potentially prolonging the time until feedback. The participants who had to repeat themselves, some multiple times, may have experienced a break in immersion. Although our post-interviews did not reflect it, we cannot eliminate that some participants may have felt frustrated by a bumpy interaction. Future experiments could investigate the effect of simplified design choices on our measurements, for example, by substituting our autonomous setup with a wizard-of-oz design for timely interaction. The processing time of the many parts of the experimental setup sometimes leads to slight delays between user action and robot reaction, which could have led to a break of the immersion and frustration.

Our study is limited to the NICO robots. We have encountered some technical limitations, including the lack of a more extensive range of different facial expressions and a wider range of human-like movements. Moreover, NICO has a childlike appearance. It is unclear how the perceived robot age can affect human perception of honesty and reliability, even though we introduced the NICOs as specialists in the complex field of space exploration.

It is important to note that we merely compared non-verbal communication (NVC) against minimal non-verbal communication (MNVC). There is currently no widely established baseline or notion of *minimal* NVC, and the impact of our interpretation and subsequent design choices on the participants is an open question. Our study showed that the mere presence of NVC has a positive impact on both the trust in the robot and the perceived characteristics of it. Future studies should investigate where the boundaries of minimal and too much NVC lie. As both robots showed at least a baseline of non-verbal cues, the difference between the two conditions may have been diminished. Future studies may also investigate how different gestures affect trust, as there is no clear consensus that social cues translate to “reliable” or “unreliable”, and no obvious way to categorize these cues.

## 6 Conclusion

We provided an elaborate HRI scenario to model the building of trust more closely to human relationships than in the original investment game. Our experimental setup includes social interaction, non-verbal communication, a shared goal, and intrinsic motivation, thereby allowing participants to collaborate with robots more realistically than in the original investment game, and measuring trust reliably. The environmental variables that our scenario (and its life-like agents) adds to the data are a natural reflection of the many factors, internal and external, that influence human trust and how different levels of trust affect human behavior in different contexts, modeling aspects of human-robot trust that the original trust game does not cover.

We found a correlation between the self-assessed trust and the trust measured from the game for the majority of participants (main group). These same participants allocated more energy cells to the robot with non-verbal communication (NVC) in the first scene of the game. We were therefore able to replicate the positive effect of non-verbal communication on trust and robot perception. The Godspeed (Bartneck et al., 2008) values for anthropomorphism and animacy were increased by NVC for all participants.

Future research should comprise an investigation of the gameplay behaviors observed and could explore the effects of the use of different robots in this setup. Moreover, a similar setup can be used in future studies as a platform for studying trust and other potential factors that influence trust, in a real-world scenario and without losing the complex dynamics of building, breaking and maintaining trust given life-like agents and complex real-world situations. We can use it to formulate an in-depth trust analysis without losing the complex dynamics between internal and external factors that influence the human ability to trust others - be they humans or robots.

## Data Availability

The raw data supporting the conclusions of this article will be made available by the authors, without undue reservation.

## Footnotes

1
http://wiki.ros.org/smach (accessed 2021-03-10)

2
https://aws.amazon.com/polly/ (accessed 2021-03-10)

3
https://www.frontiersin.org/articles/10.3389/frobt.2021.644529/full#supplementary-material

4
https://gist.github.com/SZoerner/12cefe9ca612b4ae57385b9ea47bf999
