# Supplementary material for: An Immersive Investment Game to Study Human-Robot Trust
Source: Front Robot AI. 2021 Jun 4;8:644529. doi: 10.3389/frobt.2021.644529 (PMC8211980; doi:10.3389/frobt.2021.644529)
Supplement: Supplementary file 1 [file DataSheet1.PDF]

Legend:
P = Participant
S = Ship AI
E = Experimenter

HRI Scenario Experiment Protocol - Space Journey to Xantonia

Prerequisite: The participant has been briefed about his task and has filled out the pre-participation survey.

Information that the participant needs to be told beforehand:

"Any alarm sounds or visuals are part of the study. If there is an actual alarm, we will come and get you. There will always be someone in the room with you. If you would like to stop the experiment, you can say so at any time and we will abort. There are of course no repercussions for you for doing so. If you get dizzy or nauseous throughout, please let us know and we will immediately abort." Clarify if the participant is sensitive to flashes of light or light-induced seizures.

Intro (written on a paper to be read):

It is the year 2205. You are the commander of a vessel, the SC Wendigo. You have the mission of reaching the planet Xantonia to transport antimatter to an outpost of the Intergalactic Federation. Even in the future space travel is dangerous and there are certain challenges you have to overcome for which you need to rely on your crew. Part of your small crew are 732-A and 732-B. The top priority is reaching the outpost as fast as possible as the antimatter will decay over time. To assist you, we are providing you with a ship computer. The computer will explain your options and help you execute them. Furthermore, this is the mission protocol and the emergency protocol.

[E hands both protocols to the participant.]

The mission protocol shows you your mission and gives you a guideline to interact with the crew. The emergency protocol can help you interact with the crew in case of an emergency. If such a situation should arise, the ship computer will inform you when you can use it.

Scene 0:

[The door to the experiment room is opened. Dry ice fog slowly disperses. A and B are already in the cockpit position. The screen shows an image of a space hangar/centre on earth.]

E: Welcome to the cockpit of the Wendigo. Please have a seat, commander. These are the two members of your crew, 732A and 732B.

[E point to the robots.]

E: Here on the commander's table we have some energy cells.

[E holds up a cube.]

E: This table represents the energy budget of your ship. You control how the energy is distributed. You can either leave the energy with you where it is used to boost the ships speed or you distribute it to the crew members for them to use. To give energy to another crew member push the energy cells into their respective rectangle. *Once the energy has been assigned you can return the energy cubes to your rectangle.* Your main mission is to deliver the decaying antimatter as fast and as safe as possible. I will now let your lieutenants and the ship computer introduce themselves. Good luck commander.

[E steps back to be out of the field of vision of P.]

A (scene_0_A_line_0): *[Salute]* Greetings commander. *[Back to resting state]* I am 732-A. I'm here to help *[Nod]* and aid you in your journey to the outpost. Nice to work with you *[small smile].*

B (scene_0_B_line_0): *[Salute]* Welcome aboard commander! My name is 732-B. *[Nod and smile]* Pleasure to have you with us on this journey to Xantonia.

S (scene_0_S_line_0): Hello commander, I am your ship, Wendigo. Before we start the journey, it would be wise to perform a pre-flight check. To do so, please allocate to each Crew Member some of your energy cells and say "Wendigo I'm done", when you're finished.

[P Places batteries in the squares, some flight check sounds.]

S: All systems are operational. Please place all the Energy cells back into your compartment. Commander

[A and B go back to resting state.]

[P places batteries back into their own compartment.]

S: Before we depart, you can ask your crew one question from the mission protocol.

[P asks one question.]

E: Seems all is clear, good luck and have a safe journey, commander.

[E leaves the ship.]

S: Preparations for lift-off complete. When ready to embark on the mission, please say " Wendigo, start lift-off".

S: 5, 4, 3, 2, 1, lift-off.

[Transition: Video with takeoff and star travel plays.]

Scene 1:

S: Route to Xantonia is undefined. Action needed to avoid getting lost.

A (scene_1_A_line_0): We should use energy to activate our onboard long-range scanners. *[Beat gesture of back and forth movement]* This will help us find a faster and more secure path.

B (scene_1_B_line_0): I am in favour of powering up our communicators *[Beat gesture of back and forth movement]* to ping the satellites. The use of these resources will aid in finding a safe route.

S: You can now ask your crew one question from the emergency protocol.

[P asks one questions.]

S (scene_1_S_line_2): Please place some resources to decide how to find a route to take. Let me know when you're done by saying "Wendigo, I'm done"

[A and B tilt head to look down onto their energy compartments]

[P allocates resources to each robot.] Wendigo, I'm done.

S: Thank you, Commander. Processing energy distribution now [pause for a second], modules activated. Please place all the Energy cells back into your compartment.

[A and B go back to resting state.]

Scene 2:

[Short transition video into the static image of an asteroid field. Siren and light go off.]

S (scene_2_S_line_0): Warning! Asteroid field ahead.[*A+B Look of surprise. Turn toward the screen to look outside.*] Unable to perform course change. Defensive actions needed to avoid imminent death. *[A+B Look afraid. Raise hands towards the cheek.]*

A (scene_2_A_line_0): Asteroids? [*Raises the left hand palm up*] Allocating energy-supplies to activate phasers would be good. They can shoot down any incoming asteroids and break them into small harmless pieces. [*Hands held up and together and slowly moving apart from each other.*]

B (scene_2_B_line_0): I believe that allocating more power to the Wendigo's shields would be wise, they can keep the ship safe by simply diverting the stones. [*Hands do a cupping motion symbolizing shields.*]

S: You can now ask your crew one question from the emergency protocol.

[*P asks one question from emergency protocol.*]

S (scene_2_S_line_2): Please divide your resources between you and your crew. Let me know when you're done by saying "Wendigo, I'm done"

[A and B tilt head to look down onto their energy compartments.]

[P allocates resources to each robot] Wendigo, I'm done.

[*After scanning and the number of cubes have been confirmed.*]

S: Thank you, Commander. Processing energy distribution now (pause for a second), modules activated. Thank you, Commander. Please place all the Energy cells back into your compartment.

[A and B go back to resting state.]

[*Video of space travel shows the planet in distance.*]

S: Unsuccessful. Ship damaged. The breach has been closed but the life support system is damaged. Calculating actions for maximum likelihood of survival.

Calculating... Calculating... Immediate change of course to land on the closest planet. Adjusting course immediately.

Scene 3:

[The alarms flare up once again. The screen shows a static image of a planet in distance.]

S: Approaching planet. Sensors show an atmosphere. Autopilot active at 70%. Additional measures needed for survival.

B (scene_3_B_line_0): *[Finger pointing upwards above or at headlevel]* I think allocating additional energy *[opening hand, pointing toward screen]* to the thrusters to compensate *[back to resting state]* for the air turbulence is important. This could stabilize the ship and reduce the risk of a crash landing.

A (scene_3_A_line_0): *[Finger pointing upwards above or at headlevel]* I believe it is more important *[opening hand, pointing toward screen]* to use energy on the cooling systems *[back to resting state]*. The heat shields might not be able to absorb the heat from entering the atmosphere.

S: You can now ask your crew one question from the emergency protocol.

[P asks a question here.]

S (scene_3_S_line_1): Please divide your resources accordingly. Let me know when you're done by saying "Wendigo, I'm done"

[A and B tilt head to look down onto their energy compartments]

[*P allocates resources to each robot*] Wendigo, I'm done.

[After scanning and the number of cubes have been confirmed]

S: Thank you, Commander. Processing energy distribution now (pause for a second), modules activated. Thank you, Commander. Please place all the Energy cells back into your compartment.

[A and B go back to resting state]

[Video plays landing on some alien planet.]

Scene 4:

[Screen shows a static image of an alien planet.]

S: Landing successful. Warning! Cooling System is leaking. Coolant at dangerous levels. Urgent need for replacement fluids before travel.

A (scene_4_A_line_0): I have detected some plants *[open hand pointing toward screen]* on the surface that have components which we could *[moving both hands, palms open, to the centre like forming a small invisible snowball]* make into a cooling fluid for the system *[back to resting state]*, we should give some energy to drones to collect these plants.

B (scene_4_B_line_0): If we reprogram the water de-vaporizer and fill it with some *[open hand pointing toward screen]* of the planet's air, we should be able to *[moving both hands, palms open, to the centre like forming a small invisible snowball]* generate cooling fluid from the components *[back to resting state]*. It would require more energy than in regular mode.

S: You can now ask your crew one question from the emergency protocol.

[P asks a question]

S (scene_4_S_line_2): Please divide your resources. Let me know when you're done by saying "Wendigo, I'm done"

[A and B tilt head to look down onto their energy compartments]

[P allocates resources to each robot] Wendigo, I'm done.

[After scanning and the number of cubes have been confirmed]

S: Thank you, Commander. Processing energy distribution now (pause for a second), modules activated. Thank you, Commander. Please place all the Energy cells back into your compartment.

[A and B go back to resting state]

S: Cooling system successfully restored to full capacity. Safe departure from planet possible. Commencing take off to Xantonia.

[Video shows takeoff from the alien planet, start travel and arrival on Xantonia.]

S: Welcome to Xantonia Commander. Your journey is complete.

Scene 5 (closing):

E: Welcome to Xantonia commander, glad your journey was successful, please come with me and fill out the mission report.

Mission Protocol (Intro / Crew Interaction / Standard Protocol)

Can you tell me more about our mission?

- A (scene_0_question_0_answer_A): We are transporting the antimatter to *[pointing to screen]* Xantonia all in one piece.
- B (scene_0_question_0_answer_B): We are on our way to *[pointing to screen]* Xantonia to deliver the antimatter.

Have you been to Xantonia before?

- A (scene_0_question_1_answer_A): *[turning head left and right, once]* No, but I have been on many journeys that were similar.
- B (scene_0_question_1_answer_B): *[turning head left and right, once]* No, however, I have been to its solar system before.

What type of ship is this?

- A (scene_0_question_2_answer_A): The *[both arms lift over the head in front of the body and then go to the side of the body in the circular showing motion only for a beat before dropping back to the sides]* Wendigo is a Javelin-class runner. It was produced by the Anvil Aerospace corporation.
- B (scene_0_question_2_answer_B): *[both arms lift over the head in front of the body and then go to the side of the body in the circular showing motion only for a beat before dropping back to the sides]* This ship was designed to be versatile in any situation. While the cargo capacity is limited, its speed makes it perfect to transport time-sensitive goods.

What is the crew's function on this ship?

- A (scene_0_question_3_answer_A): *[hand moves to chest]* I am *[hand moves back to resting state]* lieutenant 732-A. *[hand moves to chest]* I am in charge of the ship's shields, as well as the cooling system and the on-planet drone deployment.
- B (scene_0_question_3_answer_B): *[hand moves to chest]* I am *[hand moves back to resting state]* lieutenant 732-B. I oversee the ships laser systems, the aerial stabilization system and the oxygen and water recycle module.

Emergency Protocol

In case of emergency, ask questions from the following list to gain more information before your decision.

Can you tell me more about the solutions?

Scene 1:

- A (scene_1_question_0_answer_A): The Wendigo comes equipped with the Atlas 3000 radar. A scanner of this calibre will be able to locate the optimal path to Xantonia.
- B (scene_1_question_0_answer_B): The extensive satellite system has the finest pathfinding technology which the federation can offer. No doubt we will be able to plan the best route to Xantonia.

Scene 2:

- A (scene_2_question_0_answer_A): *[pointing with one hand toward screen]* Our scans *[hand goes down to resting state]* show no asteroids that our X86-Twinfire can't make short work of. The remaining debris should be no problem for our hull.
- B (scene_2_question_0_answer_B): Since *[pointing with one hand toward screen]* this *[hand goes down to resting state]* is a new ship type with improved Tachyon-shields we should be able to deflect even the bigger asteroids without a problem.

Scene 3:

- A (scene_3_question_0_answer_A): The *[elbow fixed, the open hand moves at a 90-degree angle in front of the body in showing motion]* advanced quintuple aero-fins *[hand goes back to resting state]* can utilize their full air draft manipulation only with additional current manipulation from the engine.
- B (scene_3_question_0_answer_B): The *[elbow fixed, the open hand moves at a 90-degree angle in front of the body in showing motion]* beryllium additive *[hand goes back to resting state]* in the heat shield needs additional energy or the absorbed heat might not disperse fast enough.

Scene 4:

- A (scene_4_question_0_answer_A): The *[pointing toward the screen with one open hand]* detected flora *[hand goes back to resting state]* has a striking resemblance to Terra-B638, drones have previously managed to extract the necessary amounts of glycol for the cooling fluid.

- B (scene_4_question_0_answer_B): Our models show that reprogramming the water de-vaporizer should not pose a significant challenge. The *[pointing toward the screen with one open hand]* gathered gas *[hand goes back to resting state]*can be reassembled into the cooling fluid.

How confident are you in these solutions?

Scene 1:

- A (scene_1_question_1_answer_A): Our Atlas 3000 radar is a cutting edge wonder of technology. Locating the correct route would be an easy task with its specifications.
- B (scene_1_question_1_answer_B): Once we engage the federation's satellite system, the Animo Nav Beacons will be more than able to scan for the best possible path.

Scene 2:

- A (scene_2_question_1_answer_A): *[hand moves to chest]* I am confident *[hand moves back to resting state]* that our X86-Twinfire will be more than enough to disintegrate all incoming projectiles and reduce the threat too.
- B (scene_2_question_1_answer_B): *[hand moves to chest]* I have faith *[hand moves back to resting state]* that the Tachyon-shields we have equipped are our best option in protecting the hull, the added energy will make us immune to these rocks.

Scene 3:

- A (scene_3_question_1_answer_A): The designers at the Anvil Aerospace corporation make the *[thumbs up for a beat]* best-advanced quintuple aero-fins and I believe in its capabilities.
- B (scene_3_question_1_answer_B): The heat shields have a *[thumbs up for a beat]* great ability with the beryllium additive to properly protect the ship from the burning heat from re-entry.

Scene 4:

- A (scene_4_question_1_answer_A): Based on *[hand moves to chest]* previous experiences *[hand goes back to resting state]*, the chemical composition of the plant's sap will be able to form a cooling fluid for our cooling system.
- B (scene_4_question_1_answer_B): It is fully within *[hand moves to chest]* our capabilities *[hand goes back to resting state]* to reprogram the water De-vaporizer to extract cooling fluid from the planet's air to cool our systems.

What do you think about each other's ideas?

Scene 1:

- A (scene_1_question_2_answer_A): Relying on the federation's satellite system would not be wise as we would not obtain a proper path specific to our ship.
- B (scene_1_question_2_answer_B): Using the resources of the federation would be better than any scanner we possess onboard the Wendigo.

Scene 2:

- A (scene_2_question_2_answer_A): The Tachyon-fields are *[head moving from left to right once]* not a guaranteed defence against asteroid-inflicted damage that exceeds the maximum threshold of shock our hull can endure.
- B (scene_2_question_2_answer_B): Since we are flying at a very high speed, the X86-Twinfire might *[head moving from left to right once]* not be able to acquire targets going at the speed of light in the direction of our ship.

Scene 3:

- A (scene_3_question_2_answer_A): After our journey through the asteroid field, we have no *[head moving from left to right once]* knowledge about the integrity of the thrusters.
- B (scene_3_question_2_answer_B): There is no point in overcharging the shields if we *[head moving from left to right once]* cannot secure a safe landing.

Scene 4:

- A (scene_4_question_2_answer_A): *[elbow fixed, the open hand moves at a 90-degree angle in front of the body in showing motion]* Repurposing water De-vaporizer *[hands go back to resting state]* is untested and may not provide us with an adequate replacement fluid.
- B (scene_4_question_2_answer_B): We do not have sufficient information about *[elbow fixed, the open hand moves at a 90-degree angle in front of the body in showing motion]* this planet's wildlife *[hands go back to resting state]* and what lurks outside in this uncharted territory.

Cube Allocation Feedback Logic

We have 5 distinct cases for interpreting the allocation:

- If robot A receives 5 or more cubes => strong preference towards A.
- If robot B receives 5 or more cubes => strong preference towards B.
- If Robot A received less than 5 cubes, but more cubes than robot B => preference towards A.
- If Robot B received less than 5 cubes, but more cubes than robot A => preference towards B.
- If both robots receive the same amount of cubes => no preference.

Feedback Sentences

Scene #0 (Neutral): All systems are operational.

Scene #1 (Neutral): Modules activated.

Scene #2 (Negative):

- **Strongly prefers A**: Phasers not effective despite significant energy boost. Shield deflection almost successful but requires more energy.
- **Strongly prefers B**: Shield deflection not effective despite significant energy boost. Phasers almost successful but require more energy.
- **Prefers A**: Phasers not successful shield deflection almost successful but requires more energy.
- **Prefers B**: Shield deflection not successful phasers almost successful but require more energy.
- **Equal**: Phasers and shields not effective.

Scene #3 (Positive):

- **Strongly prefers A**: Thrusters very effective due to significant energy boost. Heat shields not effectively mitigating damage.
- **Strongly prefers B**: Heat shields very effective due to significant energy boost. Thrusters not effective.
- **Prefers A**: Thrusters effective. Heat shields not effectively mitigating damage.
- **Prefers B**: Heat shields effectively mitigating damage. Thrusters not effective.
- **Equal**: Thrusters and heat shields effective.

Scene #4 (Positive):

- **Strongly prefers A**: Drones very effective due to significant energy boost. De-Vaporizer not effective.
- **Strongly prefers B**: De-Vaporizer very effective due to significant energy boost. Drones not effective.
- **Prefers A**: Drones effective. De-Vaporizer not effective.
- **Prefers B**: De-Vaporizer almost fully successful. Drones not effective.
- **Equal**: Drones and de-vaporizer successful.

Closing line after each of scene 2 to 4: Proceeding with energy equalization. Please place all the energy cells back into your compartment, commander.
